# Indonesia as a legal welfare state: A prophetic-transcendental basis

**DOI:** 10.1016/j.heliyon.2021.e07865

**Published:** 2021-08-23

**Authors:** Khudzaifah Dimyati, Haedar Nashir, Elviandri Elviandri, Absori Absori, Kelik Wardiono, Arief Budiono

**Affiliations:** a Universitas Muhammadiyah Surakarta, Indonesia; b Universitas Muhammadiyah Yogyakarta, Indonesia; c Universitas Muhammadiyah Riau, Indonesia

**Keywords:** Welfare, Law, Indonesia, Transcendental, Prophetic, Philosophy

## Abstract

**Introduction:**

The modern welfare state concept is based on individualistic and liberal social contracts that prioritize individual interests with liberalist, partial and non-holistic hegemonies. The welfare state concept has failed in directing citizens to achieve justice, peace, and welfare. The basic aim of the Republic of Indonesia's formation is to form a welfare country. But the Indonesian constitutional articles do not have the power to form a welfare society compared to those of other states, such as Norway, Japan, the USA, and Malaysia, whose constitutions aim to achieve welfare.

**Objective:**

This research aims to explore the formulation of Indonesia as the Prophetic Transcendental-Based Legal Welfare State. The urgency of this research is on the effort of model invention, or a new postulate on the form of the Indonesian Legal Welfare State.

**Methods:**

This is normative-juridical research with a philosophical approach to find and explore the formulation of Indonesia as the Prophetic Transcendental-Based Legal Welfare State.

**Conclusion:**

This research found that as a Legal Welfare State, Indonesia inspirits Pancasila as the moral-spiritual guidance in all developmental policies on the national law, economy, politics, and culture. Thus, the direction of the state's development is inseparable from its roots, namely the prophetic and transcendental Pancasila.

**Novelty:**

The previous researches discussed Indonesia as a welfare state only based on the determining legislation or laws. But this research discusses the philosophical aspect based on the thoughts of Indonesia's founding fathers.

## 1 Introduction

Globalization hugely influences the nation's existence as the economic controller. Economic, social, and political issues are mostly related to the nation's failure to undergo its roles well. As a legal institution, a state must not ignore the people into a position of incapability to fulfill their basic needs in providing basic social services (Setiawan, 2011).

Ohmae (1995:11–17) explicitly announces the end of the ‘nation-state’. The declining power of a nation is definite in the rise of the global economy. As an institution, the state is no longer perceived as an important agent as the reality of a borderless world describes a vital consequence; the diluting ethics of border identities (nationalism, religion, community). These identities used to be the main guidance, but they fade and collapse due to the surge of globalization (Ohmae 1995:11–17).

The case is not merely the decline of 'the nation-state', as Mishra contends that globalization has hindered the nation's capacity to have social protection (Ramesh, 2000). Moreover, Fukuyama (2005:xix) describes the diminishing roles and strategic functions of nations that worsen poverty and social gap – and possibly igniting social conflicts and civil war. That simple picture explains the immense paradox of the welfare paradigm. The transformation of the capitalistic paradigm creates a big leap into neoliberalism. Subsidies as the assurance of basic need services are assumed as inefficient. Neoliberalism incorporates capitalist economy and free trade systems as tools to create a market syndicate. According to John Perkin's term of corporatocracy syndicate, it aims to gain profits by promoting corruption, collusion, and nepotism from the third nations, and it operates like a mafia syndicate (Perkins, 2008:12–13).

The failure of the welfare nation applied by the economic and political policy experts due to the forceful hit and wave of the economic crisis should become a lesson to review the legal wrongs of the state. In this sense, Indonesia should learn from these occurrences as it is difficult for this country to exit the vicious circle of an economic crisis that led to the fall of Soeharto's presidency up to the failure of the Reformation government to make Indonesia a welfare state.

Fundamentally, the purpose of the Republic of Indonesia's formation is to create welfare for its people. Typically, Indonesia aims at creating a welfare nation. The national development's objective is to increase social prosperity, including in the aspect of economic growth and in fulfilling the basic social and economic needs of all citizens, so that they may at least live at the minimum living standard. That is the nation's moral ideal as created in the formation of this country. It is the compass or the direction that the Indonesian nation hopes to achieve.

The number of welfare articles in the Republic of Indonesia's 1945 Constitution and its amendment is more numerous than those of other countries with relatively higher human development index (HDI), such as Norway, the United States of America, Malaysia (three articles), and Japan (one article). Indonesia has 14 welfare articles in the 1945 Constitution, but it has not yet created a prosperous society. In Indonesia, the foundation of the welfare state is influenced by religious values. Even though the majority of Indonesians are Muslims, Indonesia is not an Islamic State. It is a state with multiple religions, ethnicities, and cultures and it does not ignore the influence of other religions and ethnicities. This Transcendental-Prophetic paradigm is derived from religious thought with interconnection and inter-disciplinary paradigms with other transcendental and cultural values, that aim to analyze the Indonesian welfare state.

The people have the legitimacy to demand the state to fulfill its responsibility. Therefore, they can demand the rights written in the constitution. This issue becomes the background for the writers to reconstruct the prophetic-transcendental-based Indonesian legal welfare state so that the nation will become fully responsible for creating essential welfare.

## 2 Research objective

There has already been much research on Indonesia as a welfare state. But they have only discussed the constitutional regulations or the legislations which determine Indonesia as a welfare state. Those researches discuss Indonesia as a welfare state in the legal-formal perspective and how Indonesia has not yet prospered even though many formal regulations have stated it as a welfare state. In reality, the poor people's rate of welfare is still very low. The efforts and services to eradicate poverty are still centralized in cities. The poverty rate is still high. Thus, the people of rural areas have not yet obtained adequate welfare (Dewanta, 2005:67).

This research especially discusses the philosophy and the bases on why Indonesia chooses to become a welfare state, especially in the visions of Indonesia's founding fathers. The Indonesian founding fathers are 68 people who strived for the Indonesian nation's independence from colonization. They have roles in formulating the post-independence form of the state. They came from various backgrounds of education, region of origin, ethnic, and/or religious backgrounds (though most of them are Muslims because the majority of Indonesians are Muslims) (Sularto and Yunarti, 2010).

Through this research, the writers hope to find the link to understand the philosophical-transcendental welfare state as envisioned by the founding fathers. In the end, this research is hoped to bring clarity on the understanding and the thought in developing Indonesia as a welfare state.

The basic idea of the welfare state as formulated by the Indonesian founding fathers is different from that of other countries. It departs from the religious beliefs of the majority of the Indonesian people, with an agreement and an understanding from various ethnic and religious representatives. Muslims accepted the idea from other representatives to erase the phrase, “For the Muslims to implement the Islamic Sharia,” and the representatives from other ethnicities and religions accepted the idea that Indonesia must not become a secular country. They agreed to form Indonesia as a multi-religious and multicultural country. As citizens, the founding fathers acknowledge this condition. This ideal vision is then translated into the constitutional regulations or policies in achieving welfare. These regulations and policies are not yet able to bring the welfare state to reality, thus the researchers are interested in carrying out a study on this condition.

## 3 Materials and methods

This research uses the juridical-normative method with the prophetic-transcendental philosophy approach. According to Gulen (2006:103), the transcendental approach will result in the ultimate intelligence, which is carried out by breaking through the formalism boundaries (exciting rule) and transcendence, to obtain a new way of thinking that is close to the ultimate truth. According to Shiddiqi (1993), the transcendental approach departs from the paradigm as well as *hudan* (guidance of wisdom) and *rakhmah* (peace). According to Absori et al. (2018:12) in this perspective, knowledge is not only for the sake of knowledge, but it must be beneficial for the good of the public. This means that to reach an understanding of Indonesia as a legal state of welfare in the transcendental-prophetic perspective, we must find and identify Indonesia's welfare laws in the transcendental-prophetic philosophical approach framework (Dimyati, 2014).

This is juridical-normative research with a transcendental approach. It means that the data on Indonesia as a welfare state is inventoried. These data include thoughts, books, and statements of Indonesia's founding fathers on the philosophy of Indonesia as a welfare state. The inventoried data are then processed and analyzed using the descriptive, dialectic, and interpretative methods (Sidharta, 2011:145).

Epistemologically, the method with the transcendental approach with the interdisciplinary and interconnectional paradigm describes the worldview of new science that is more open. It can open scientifically accountable and forward-looking dialogs and cooperation. The interdisciplinary and interconnectional paradigm between factual-historical-empiric sciences is social sciences and natural sciences (*Hadlarah al-Ilm*) as well as ethical-philosophic scientific culture (*Hadlarah al-Falsafah*) (Sutarto, 2017).

According to Amin Abdullah, as quoted by Sari (2019:114), this method exhibits great contribution to sciences related to the humanity-contemporary approaches, such as hermeneutics, contemporary linguistics, natural sciences, by re-discovering the power of scientific treasure. These methods, approaches, and paradigms are compared. Then, they lead to a choice of integrative and interconnective scientific formats that can lower the intensity of interdisciplinary conflicts.

Responding to this reality, as quoted by Waston (2016), M. Amin Abdullah states that all scientific structures, such as religious studies (including the study of Islam and other religions), social studies, and humanities, as well as natural science cannot stand individually. When certain studies claim to be able to stand alone and to resolve their issues individually without any support or contributions from other studies, this self-sufficiency will sooner or later evolve into narrowmindedness – to not mention particularity fanaticism of scientific disciplines. Thus, cooperation, greetings, corrections, and connectedness between disciplines will further help humans in understanding the life complexities they experience and resolve the problems they face (Abdullah, 2006:223).

The writer uses the descriptive analysis, as this study is aimed to obtain a systematic and objective description of Indonesia as a Legal Welfare State, its characteristics, and also the links between the elements which form the Legal Welfare State (Chamsyah, 2008:201). The writers use the juridical-normative method with the transcendental approach and the interconnection and interdisciplinary paradigms considering that Indonesia as a legal welfare state is not only purely based on regulations, but it is inspirited by the transcendental-prophetic influences, where Indonesia is a nation whose majority of its population consists of Muslims. After obtaining the results of this analysis, the writer then carries out the interpretative analysis to find the meaning behind the results. Then, the writer finds the results on the prophetic-transcendental philosophic basis of Indonesia's founding fathers.

This research uses the following coherences:

a. *Vestehen* (Understanding)

The method of understanding a research object, for instance, a thought, using insight and empathy. This is sourced from M. Amin Abdullah's thought on the fundamental structure of epistemology.

b. Internal Coherence

This method gives an accurate interpretation of a figure's thoughts, so that all concepts and aspects may be seen from its harmony between one and another. Then the fundamental core thought is determined from the central topics of that figure. Then, it is analyzed according to the logical-systematic structure in its thought development, and compared with the style and method of thought. In this case, the writer uses the thought of Amin Abdullah as the research material.

In the tradition of transcendental-prophetic thought, there are at least three forms of knowledge of epistemology, namely: *bayani, irfani,* and *burhani*. The *bayani* mindset emphasizes *qiyas* or equation (*qiyas al-illah* for fiqh and *qiyas al-dalalah* for kalam) instead of *manthiq* (careful calculation). Because of that, it is no wonder that this pattern of thought emphasizes textual epistemology. Meanwhile, the *irfani* epistemological pattern is sourced from intuition instead of literal texts. The main source of knowledge in *irfani* is direct experience. The experience mentioned here is addressed to deeply profound inner experiences. This last epistemology in the *Isyraqi* tradition of the East is known as *al-Ilm al-Hudhuri* or preverbal, pre-reflective consciousness or prelogical knowledge which are familiar in the Existential tradition of the West.

The usage of the interdisciplinary and the interconnection paradigms as well as methods and approaches make it possible for the authors to understand the research more profoundly. Thus, the writer can extract the philosophy from all the analyzed data. Then, the writer dialects the philosophy of the legal welfare state using the prophetic-transcendental approach. The researcher also dialects the founding fathers’ concept of the legal welfare state and the gap between their thoughts and the application of the legal welfare state.

## 4 Results and discussion

### 4.1 The construction of the legal welfare state

As the main theme of this article, the legal welfare state concept requires further explanation. According to writers, the legal welfare state concept is the basis of thoughts in a developing socio-economic system that incorporates justice values as its soul. Manan (1996:9) explains that the legal welfare state concept is as follows, “A nation or a government is not only the guardian of security or conduct but also the main authority in creating social justice and public prosperity.”

According to Muchsan, the legal welfare state has some characteristics. It reasonably directs people to a prosperous life; it provides the best and the maximum services for society. In the social aspect, it demands the achievement of social justice and public welfare. Without such things, it will be impossible to create social welfare. Based on those characteristics, there are two definite phenomena in a welfare state, including (1) vast government intervention in the social life aspect and (2) the use of the discretionary principle in applying governmental functions (Muchsan, 1992:4–5).

During the process of achieving public prosperity, the emerging legal welfare state concept is introduced by Otto Bar as cited in Muslimin (1982:87): The Indonesian modern legal state turns into a nation with multi-cultural characteristics (*culturstaaf*) or a welfare state (*welvaarstaat*). This causes Indonesia to also acknowledge the customary laws that apply in multi-ethnic and multi-cultural societies. It is hoped that the application of the customary laws will increase harmony in Indonesia as a requirement of the welfare state.

According to Iver (1950:4), a nation is not only perceived as an instrument of power. Even more, it comes to be an agency of services. Such pragmatic view then develops the conception of a welfare state or modern legal state or a legal material state, that indicates the following characteristics: a) in the legal welfare state, the fundamental issue is the assurance of the people's socio-economic rights; b) it prioritizes the consideration of efficiency and management instead of power share for political orientations, therefore the executive roles are greater than that of the legislative; c) the principles of administrative law has greater authority in regulating the social economy and in imposing certain responsibilities to people.

From the above explanation, the country has a vital and superior position in creating public welfare and social justice. In many works of literature, such conception of nations refers to a state of social services, an agency of services, or social *reehtsstaat*. Such conception of the modern legal state requires the government or the national conduct to be based on the law. The country also possesses wider roles, duties, responsibilities to bring prosperity for people (Panjaitan, 2012).

To sum up, the duties of a county *in case* government is to formulate every article to create public welfare, so that the people can see that law plays its role in developing the people's welfare. In this case, Raharjo (2006, 9–11) further asserts that “the law must be blissful.” The welfare state concept in the Constitution and the national principle named Pancasila is crystallized from the desires and the efforts in formulating that common ideal. In the welfare state concept, the textual and contextual reading of Pancasila in its formulation process means finding knowledge on how the Indonesian welfare state is designed. The Pancasila conceptualization starts from the formulation and the attestation processes at the first trial of Badan Penyelidik Usaha Persiapan Kemerdekaan Indonesia/The Investigating Committee for Preparatory Work for Indonesian Independence (abbreviated BPUPKI).

The formulation of Indonesia as a legal welfare state is a progressive thought of the founding fathers. It resulted in an impressive formulation of the legal welfare state. These founding fathers made efforts to adopt both religious-prophetic and secular ideas and thoughts, between the legal state and the welfare state concepts, by considering Indonesia as a nation with multiple ethnicities, cultures, and religions, as stipulated in Pancasila or the Five Principles and the 1945 Constitution.

### 4.2 The formulation of the Indonesian legal welfare state

Indonesia aims to become a legal welfare state. All used terms orient to public welfare. Its founding fathers apply the ‘just and prosper’ terms in the second paragraph of the Preamble of the 1945 Constitution. The other terms are “public welfare” and “social justice” in the fourth paragraph of the Preamble. Section 3, Article no. 33 of the 1945 Constitution is in line with the terms “social justice” and “public prosperity” (Suhardin, 2007). The formulation of Indonesia as a welfare state cannot be separated from the formulation of Pancasila and the 1945 Constitution that were conceived by the Committee of Nine (Panitia Sembilan), where Indonesia is a welfare state. It is based on Pancasila's fifth principle, namely, “Social justice for all Indonesians” (Prasetyo, 2011:3).

The Indonesian legal state cannot be defined as an absolute individual legal state nor is it a liberal legal state. It can be recognized from the definite indispensable relation between nation and religion. National freedom in the Indonesian legal state relates to the assurance of the freedom of religion. On the contrary, there is no place for atheism nor anti-religion propaganda in Indonesia. Indonesia holds the characteristics of community (*gesellschaft*) and plurality. It embraces the principles of kinship, devoutness, and togetherness.

The root of a state structure can be traced from the history of the nation. The characteristics and identity define the basis of the nation and structure in the constitution. It can be seen from one of basic consensus in the constitution, namely the agreement on the society's general goals or the general acceptance of the common philosophy of government (Maladi, 2010).

In BPUPKI's trial, the participants deliver their opinions on the welfare state, which is now written in the fifth principle of Pancasila, namely “Social justice for all Indonesians” with the various idealized formulations. Based on the Committee of Nine's formulation, the principles of a welfare state are written in that fifth principle. It provides guidance in formulating the Indonesian Constitution (Alfitri, 2012).

Thus, the welfare state principle becomes an important part and a spiritual atmosphere of Pancasila and the 1945 Constitution. The welfare state commitment feels strong; it emphasizes the participation and sovereignty of the people. Indonesia's welfare state principle is indeed manifested as the national principle, namely Pancasila and the formulation of the 1945 Constitution (Pasha et al., 2002:54).

According to Ringga as cited by Mahfud (2007) Pancasila is a prismatic concept that assimilates the best elements from concepts with contradictory main elements. In this sense, Pancasila involves transcendental and particular values of various indigenous cultures of the present-day Indonesian archipelago, that have existed among societies for centuries. The prismatic conception can be overviewed from the following points: *first,* Pancasila involves transcendental values from individual and collective perspectives. In fact, as individuals, humans own rights and freedom along with their responsibility as social creatures. *Second,* Pancasila follows the view of a religious nation-state; it is neither a religious state nor a secular state. The nation must protect and maintain all religious adherence. From the historical point of view, Indonesia follows the path of *Rechtsstaat* or civil law because this country has been colonized by the Netherlands for centuries. However, if the civil law concept is applied, possibly, it will not bring happiness to Indonesians.

The law will move slower than the dynamics of Indonesian society. It is due to the plurality and the diversity of Indonesian society. If it is compared to the implementation of the Rule of Law in the UK, the British society is born from one line of ancestry, so there is a little difference in culture. Plus, there is also the kingdom as the symbol of unity. If this is applied in Indonesia and if liberalism is promoted, it will disunite this country (Hamzani, 2014).

Indonesia needs a typical and a particular concept of legal state. This nation is derived from the indigenous values of Indonesia; the values are not transplanted from other nations. As stated by Carl Frederich von Savigny, as *Volkgeist* (the soul of the nation), the law is a representation of the society's law awareness (Tanya et al., 2010:103). While the majority of Indonesia's founding fathers consist of Javanese and Sumatran elites Sumatra and Java, there are also those from Borneo, Sulawesi, and other places.

Surprisingly, the majority of Indonesia's founding fathers from Java and Sumatra, like Supomo (Java), Soekarno (Java-Bali), Hatta (Sumatera), and Sjahrir (Sumatra) emphasized and sought to blend traditional indigenous and modern theories of state with a modern structure and multicultural indigenous values from local social structures, and mysticism (transcendentalism) without discarding their inspired Islamic values (Taylor, 2003:194). This can be traced from the First Principle of Pancasila, namely, “Belief in the Almighty God,” which is influenced by Islamic values, but can be accepted by other beliefs. With regards to the aspiration from non-Muslims from Eastern Indonesia, the founding fathers agreed to change the original phrase that is “For the Muslims to implement the Islamic Sharia,” to become “Belief in the Almighty God” (Effendy, 2003).

As a legal product, the Constitution must be explored from the resources of Indonesian plurality, cultures, beliefs, and values. In response to the diversity of cultures and religions, the Indonesian government inserted inclusiveness into the official motto and doctrine of the country, namely “*Bhinneka Tunggal Ika*” or “Unity in Diversity” that emphasizes diversity (Cribb and Brown, 1996:7–8) and Pancasila, derived from the Sanskrit (“*panca”* meaning five and “*sila”* meaning principles) was crafted as the national doctrine. The principles of Pancasila, invented by Soekarno in 1945, are broadly treated as the basis for Indonesian politics, nationalism, and statecraft (Budiman, 1990:19). As the official philosophical foundation of Indonesia, Pancasila was intended as a way to unite the new nation and to ensure its political stability. Pancasila is credited with helping to smooth over differences between groups who had competing visions for the archipelago, regardless of their ethnicity or political beliefs (Mosher, 2008:110).

In the domain of philosophy, Pancasila and the constitution is basically aimed to provide prosperity, protection, assurance, and justice to all people. However, the law in this country experiences what we call the ideological poverty of Indonesians that substantively omits the intrinsic soul of the law itself. This is because the institutions that have the authority to dictate the law have ignored references that can be acquired from relations and dialogues of legal philosophy and domestic culture in general (Dimyati, 2015:11).

This means that Indonesia is only a welfare state in the legislations or the law, without any true manifestations. What is currently happening shows that Pancasila, as the ideals of Soekarno and the other founding fathers, have philosophies and morals that are nationally and globally adequate with the current condition. This regards the moral principles and philosophies, such as the appreciation for human rights, the efforts to bring prosperity to the citizens, and the legal state. But practically, the legal welfare state cannot be truly manifested as there are misuses of power, corruption, human rights violations, and even authoritarianism from the Republic's leaders.

Simply put, the founding fathers propose an alternative model that is more appropriate with the multicultural nation, so that the mainstream law will not be dominated by Western thoughts. At least, the Indonesian law's construction can start from several things; *first,* the empowerment of a legal system with the basis of Indonesian values. This process is ignited by the development of a system that is mixed with local wisdom as the basis in formulating constitutional regulations. *Second*, justice-based law enforcement exists in society.

The law enforcers often ignore the social justice aspect by creating controversial and discriminative policies (Dimyati, 2015:14–15). The reconstruction of Indonesian law is an agenda that must immediately be implemented. As a resolution, this research proposes the formulation of the Indonesian legal welfare state through several frameworks of thought:

*First,* based on the constitution and the law, Indonesia follows the welfare state ideology*.* This idea can be overviewed in paragraphs II and IV of the 1945 Constitution's Preamble, article 27 section 2, article 28B section 1, 28C section 1, 28D section 28H section 1 and 3 of the 1945 Constitution, article 31 section 4, article 33, article 34, the Act no. 40 of 2004 on the National Social Security System.

*Second,* the fact that Indonesia follows the welfare state ideology is confirmed and consolidated in the Constitutional Court Decree No. 50/PUU-VIII/2010 that was amended into the Constitutional Court Decree No. 007/PUU-III/2005. It states that Indonesia's welfare state ideology (vide Article 34 of the 1945 Constitution) is supported by the National Social Security System developed by the act of the same name (Alfitri, 2012). The purpose of the constitution is to provide a system that protects all citizens and that empowers the weak and the disabled based on values of humanity, as stated in the Constitutional Court Decree No. 50/PUU-VIII/2010 that was amended into the Constitutional Court Decree No. 007/PUU-III/2005.

*Third,* from the construction point of view, during a BPUPKI (Badan Penyelidik Usaha-Usaha Persiapan Kemerdekaan Indonesia or The Investigating Committee for Preparatory Work for Indonesian Independence) meeting on June 1^st^, 1945, Soekarno stated in his speech that people need to be prosperous. If we are truly considerate of the Indonesian people, we should accept the principle of *sociale rechtvaardigheid* (social justice, or the fifth principles from Pancasila), that equality must not merely be applied in politics, but also in the field of economy to reach social welfare (Azhary, 2003:97–98).

Furthermore, Yamin (1995:31) explains that Indonesia is a legal state (*rechtsstaat government of laws)* where police and soldiers guard the government and maintain justice. It is not an authoritative country (*rechtsstaat*) where power, weapons, and physical potentials govern. Tamanaha (2004:92–103) views social welfare as, “Substantive equality, welfare, preservation of community”. Iver (1950) gives a perspective related to the legal welfare state, that the principle is in the guarantee of the people's socio-economic human rights.

Padmo Wahyono asserts that law is a tool or medium to manage national life and conduct and to provide social prosperity. The 1945 Constitution explains that Indonesia recognizes the existence of unwritten laws apart from written ones. From the perspective of the kinship principle, there are three functions of law, namely: 1. to uphold democracy; 2. to bring justice as stipulated in Article 33 of the 1945 Constitution; and 3. to implement just and civilized humanity based on the belief in God Almighty (Azhary, 2003:97–98). Then, Muchsan (1992:4–5) the welfare state has some special characteristics. It reasonably directs people to a prosperous life; it provides the best and the maximum services for society. Without such things, it will be impossible to create social welfare. According to Sardar (2001:66–67), from the formulation of the Indonesian legal welfare state above, it can be summarized that the Indonesian welfare state concept intends to create social justice.

Even though Indonesia aims to become a welfare state, reality shows that it is currently a developing country. It faces many social issues, such as the high rates of poverty and unemployment, unhealthy environment, human rights violations, the low rate of education, etc. These conditions lead to ignorance and the people are prone to diseases. Thus, it is clear that the Indonesian government is still unable to act accurately nor correctly upon the welfare state concept as mandated by the 1945 Constitution (Hadiyono, 2020).

Even though juridically-formally, the constitution explicitly states the strong will and efforts to create the true Indonesian welfare state, facts show that there are many cases in massive scopes. Some policies may delegitimize Indonesia as a welfare state (Suharto, 2016:15–16).

It cannot be denied that Indonesia is still unable to reach the condition as a welfare state as idealized by Pancasila and the 1945 Constitution due to corruption and systematic legal violations, including human rights violations. There are news reports on corruption and violations of law almost every day (Davidsen et al., 2007:153).

The massive corruption cases have exceeded the law enforcers’ case-handling capabilities, as even though many corruption cases have been investigated and judged in court, the fabrication of corruptors through the corruptive system is much more massive. Worse, corruption is not a mere individual criminal case, but it involves networks, family members, and power elites. More than ever, they are involved in an advanced, organized, and consolidated modus operandi (Widjodjanto, 2012).

Indonesia seems to only ideally be a welfare state, but in reality, there are many social cases as mentioned before. The welfare state concept and laws are no longer effective when applied in statecraft practices. Pancasila seems to only become writing on the ideal welfare state that is not applied by bringing prosperity to the people, providing social justice, nor by respecting the law and human rights.

### 4.3 The prophetic-transcendental-based Indonesian legal welfare state

The transcendental paradigm can extensively be seen in the forms of religious values, ethics, and morality. The issues of those values can be confirmed by the issues of knowledge development, social, culture, economy, and law. In modern society, there are crises in creating the meaning of life (Absori, 2015a:1).

Zohar and Marshall in “*Spiritual Intelligence, The Ultimate Intelligence”* criticize the failure of Western civilization by introducing the spiritual quotient approach for ultimate intelligence. It gains by hovering over the existing rule and transcendence. The spiritual quotient (SQ) is the tool for humans to develop various new perspectives in life. They become capable of finding a wider horizon in this small world and feeling the existence of God without meeting Him. SQ is also guidance for humans during the condition of order and chaos, giving intuition above meaning and values.

SQ is outside the bindings of rulings and it is not contextual either. However, it is eager to see beyond the situation in the effort of seeking truth, meaning, or deeper values. SQ does not diminish IQ potentials nor the emotions of an individual, but it strives to improve their quality to achieve the ultimate intelligence (Absori et al., 2015b:vi-vii)*.*

The term “prophetic” in this article means: (1) of or pertaining to a prophet: prophetic inspiration; (2) of the nature of or containing a prophetic perspective related to the legal welfare state that the principle is in the guarantee of the people's socio-economic human rights. Wahyono asserts that law is a tool or a medium to manage national life and conduct. It also provides prophetic writings; (3) having the function or the powers of a prophet, as a person; (4) predictive; ominous: prophetic signs; prophetic warnings (Wikipedia). The term “prophet” is derived from the word *naba’* in Arabic, meaning news, tidings, story, and tale (Rahardjo, 1997:302)*.*

In Arabic itself, according to Ibnu Manzur, the word “prophet” correlates to the root word *al-nubuwah, al-nabawat,* and *al-nabi.* It means high land, road. *al-anbiya’* means a path for guidance and a right person due to his abilities (Asy'arie, 1999:1). The word *nubuwah* means prophecy. In Al-Qur'an, the word “prophet” and its derivatives such as *an-nabiyyuun, an-nabiyyiin, al-anbiyaa*, *an-nubuwwah* are mentioned 65 times (Roqib, 2011:47–47).

In Al-Qur'an, the words prophet (*Nabi*) and messenger of God (*Rasul*) are interchangeable. To differentiate the meanings, the *Ulama* (the Islamic priest or Islamic preacher) check the contextual meaning. From the root word, the term of *nabi* relates to their capability to accept divine revelation (Muqowim, 2001). It is supported by The Concise Encyclopedia which mentions the meaning of prophet as the one that carries out the prophecy duties in the framework of current revelation (Glasse, 1989:342). According to other religions, the prophet is people who received revelation or was sent by God to warn the people. One of the descriptions on the term of *nabi* and *rasul* in Al-Qur'an is explained in verse al-An'am (6): 89, meaning: "Those are the ones to whom We gave the Scripture and authority and prophethood. But if the disbelievers deny it, then We have entrusted it to a people who are not therein disbelievers” (Muqowim, 2001).

People in Indonesia believe that a prophecy is the highest gift for humans from God. A prophecy becomes the evidence of the spiritual aspect's superiority compared to other people. A prophet is similar to a branch of God in the mortal world. He has great intellectuality beyond other creations.

Further, the prophet is an ideal living creature (Gulen, 2002:97). Ordinary people will not obtain prophetic knowledge. *Nubuwah* (prophecy) is a title and a recognition that cannot be pursued, as it is given by Allah to chosen humans that have achieved *insan kamil* (possessing practical and theoretical thoughts) by transferring revelation. Therefore, the function of a prophet in society is basically similar to the function of consciousness inside the human heart as the mechanism of internal control to maintain life balance and to safely achieve spiritual life purpose. The support of spiritual interests will confront the tendency to control desire and to move towards transcendence (Madhkour, 1993:164).

From the socio-historical perspective, the existence of a prophet is the result of the transcendental dialectic and immanency process. The rise of prophets as moral role models and spiritual gurus becomes a law of the society's life history, that internally will arrive to become the power to maintain balance and sustainability of the social life. Based on the above description, the term “prophetic” basically relates to an ideal prophet with all prophecy characteristics. In this circumstance, if the term “prophetic is used in other entities, it should fulfill the characteristics of the prophet. Based on such understanding, generally, the term “prophetic” can be defined as follows:

“An entity that strives to prepare and put himself to be able to read and accept the divine messages and obtain wisdom from them, then to implement them in real-life situations, to benefit himself, the society and the universe”.

The first part of the above definition is “*strives to prepare and put himself to be able to read and accept the divine messages”,* based on the consideration that the recognition of prophetic quality is only performed by Allah (God). There is no room for prophet creation beyond Allah's recognition because the prophetic quality is a gift and a blessing from Allah to the chosen humans. It cannot be pursued as knowledge, spiritual training (*riyadloh*), and human compliance.

Then, the second part is *“obtain wisdom from them.”* The word “wisdom” is articulated as the capability to determine places and limits of knowledge. Wisdom becomes the final result that is gained from the use of remembrance (*dzikir*) and the mind in reading and in accepting the divine messages (Syamsuddin, 2012:300).

The third part of the phrase is “*then to implement them in real-life situations”* which means the reading and the accepting processes of divine messages. It is not sufficient to only perform in the level of thoughts and internalization. Further, it demands implementation in the social life, involving oneself in the social engineering process. It changes the world into a better place where he is involved and put to lead.

The last part of the phrase "*to benefit himself, the society and the universe,”* depicts the real purpose of a prophecy, namely to inspire virtue and to create justice inside oneself as an individual creature. If this level has been achieved, one will be capable of providing virtue to society and the universe. The meaning of “virtue” in this context is the capability to invite humans to acknowledge God and to move closer to Him and create justice and equality in society.

Pancasila is the *grundnorm* (basic norm) or the *staatfunda-mentalnorm* (nation fundamental norm) in the Indonesian legal welfare state. It is escorted by ethic and ethic-prophetic purposes (historical activism, transcendence, humanization, and liberalization). Pancasila also can be perceived as a social philosophy, a national perspective towards social phenomena. Social philosophy can define the social theory. *First,* the principle of religiosity can be defined as the social theory of positive pluralism; instead of our own religion, there are other religions to consider. Each adherence must uphold its own religion. From the social theory of pluralism, the result is the tolerant approach to religious derivatives. The application of Islamic law in Aceh province, Indonesia, must be viewed from the pluralism social theory perspective. It is a result of high politics from the peace agreement between GAM (Gerakan Aceh Merdeka or Aceh Independence Movement) and the Indonesian government to implement *Qanun* (Islamic Law in Aceh).

*Second,* the principle of humanity is the guarantee of justice and civilization. Freedom is limited by other people's rights. Therefore, a legal setup is required. *Third,* the derivation of the third Pancasila principle, “The Unity of Indonesia,” is cultural democracy and cultural pluralism. It is different from ethnocentrism that tenaciously maintains one's identity and refuses other cultural values, melting the components into a new identity. In this context, pluralism means that each ethnic group upholds its group's identities; but in some aspects, there are similar identities. Pluralism means that all areas, traditions, and cultures deserve to be conserved and developed. *Fourth*, the derivation of the principle of “People's sovereignty” is an objective of the country (technical and simple nation). It shows that the nation is the protector (*ambaureksa*) and the mandate-holder instead of the all-powerful. Likewise, *Fifth,* the principle of “Social justice” can define sociologist nationalism that recognizes the layers of a nation, and that some of them have not yet gained any benefits from the greater layers.

Social justice is a social philosophy that has been descended into social theory. The public economy is a new paradigm as the previous economy is capitalistic (Absori et al., 2015b, 316–317). A country is a legal, political, social, and cultural structure. Therefore, we allow the observation of the cultural characteristics of a nation. Then, a legal country is insisted to perform cultural displays as it is envisioned by the Indonesian Constitution. By doing so, the law should not eliminate the effort of bringing happiness to the Indonesian people, as mentioned in the aim of the constitution.

The scrutiny of the constitution results in important moral values that present and develops “a nation with robust commitment to bring bliss to the people.” All aspects in Pancasila describe these morality commitments. Some principles of Pancasila, namely: Belief in the Almighty God; just and civilized humanity; democracy guided by the inner wisdom and the unanimity arising out of deliberations among representatives; and social justice for all Indonesians oblige the nation and the government to take the mandate as duties and morality values. The meta-rational principle of “Belief in the Almighty God” is a unique character and is the reason for Indonesians to have materially and spiritually happy people.

Meanwhile, the characteristics of the Indonesian legal welfare state with the prophetic-transcendental basis are as follows: a). It upholds Pancasila as the spirit and the soul of the national movement (moral-spiritual). Any intended developmental policy must be inspired by Pancasila. Similarly, visions of economic, political, and cultural developments are inseparable from the Pancasila principles, b). There is a close relationship between religion and nation (socio-religious). It is indispensable because Indonesia is neither a liberal country nor a communist one. It is significantly related to the history of Indonesia that gained independence with the spirit of religion, c). It takes the stance of the divinity of the Supreme One (prophetic-transcendental), d). It guarantees social welfare and justice for all people, e). It promotes the freedom of religion and the practice of religion (liberation-humanization), f). Blissful laws.

The characteristics of the Indonesian legal welfare state as mentioned above, in its correlation with the prophetic-transcendental concept, can be defined in the scope of national life, because it contains universal values as its intrinsic meaning. In the context of Indonesia, prophetic transcendence is part of the fundamental conception of the “Belied in the Almighty God” principle, to create prosperity for Indonesian people (Absori, 2016). The upholding of Pancasila as the soul and the spirit of a national movement (moral-spiritual) is to characterize the policy of national legal development. Therefore, the nation's economic, political, and cultural developments apply Pancasila as the soul and the spirit of the movement, instead of incorporating other values, transplanted from other cultures and nations.

Pancasila with its five principles: (1) Belief in the Almighty God; (2) Just and civilized humanity; (3) The unity of Indonesia; (4) Democracy guided by the inner wisdom and the unanimity arising out of deliberations among representatives; and (5) Social justice for all Indonesians manifests the universal values of prophetic-transcendence in each of their principles. The first principle manifests spiritual development and religious adherence to practice rituals and beliefs based on each citizens’ own religions.

Then, the prophetic values in the second to the fifth principles are efforts to acknowledge human rights, including a reasonable, just, and civilized life standard. Thus, people mainly find civilized justice in the protection of human rights. Interestingly, in Indonesia, even though human rights are mentioned in the second Pancasila principle, namely, “Just and civilized humanity,” in reality, human rights are still not fully respected (Lestari, 2019). There are violations of human rights, such as the disbandment of demonstrations using violence (Aprilia, 2020:71–72) or the ensnaring of people who criticize the government using the Law Electronic Information and Transaction (Rahmawati et al., 2021). The cabinet misuses power by corrupting and bringing loss to public rights, such as the case of Covid-19 pandemic social aid corruption (Oktarina and Cayo, 2019:68–81) and also the case of the Bank of Indonesia Liquidity Aid. Another fundamental weakness is that severe human rights violations are not legally resolved, such as the cases of Tanjung Priok, cases of chaos in Sampit, Ambon, and Maluku, the 1998 case, and also the human rights cases during the military operation in the Aceh provincial area (Hardianti et al., 2016). All of these cases are unresolved as the perpetrators are elites who possess wealth and power.

The unresolved corruption or human rights violations cases and legal processes show that the Indonesian government ignores the founding fathers' philosophical reference to the welfare state. The second principle is “Just and civilized humanity," but ironically, severe cases of human rights violations happen and are left unresolved. The fifth principle is, "Social justice for all Indonesians," which is ironic as corruptors are imposed with similar punishments as that of common theft, even though the former massively brings loss to the public. Then, even though the fourth principle states, “Democracy guided by the inner wisdom and the unanimity arising out of deliberations among representatives," the government actually applies authoritarian governance. Some officials keep their positions for a lifetime, and the people's representatives misuse their power by only agreeing to the policies which advantage themselves and their group.

The principle of “The unity of Indonesia” maintains the life of ethnicities and local norms in Indonesia. The principle of “Democracy guided by the inner wisdom and the unanimity arising out of deliberations among representatives” is the process of thought, political, and education transformation from old-fashioned and resistant citizenship into civilized and modern people. The principle of “Social justice for all Indonesians” becomes the government's strategy to fulfill the basic welfare state has not been reached.

## 5 Conclusion

The concept of a legal country (typical and particular) in Indonesia is labeled by the writer as an Indonesian legal welfare state. This is a concept that is derived from indigenous Indonesian values and is not transplanted from other countries. It is the manifestation of the legal awareness in society as *Volkgeist* (the soul of the nation). This paper uses the juridical-normative method with the transcendental approach and the interdisciplinary and interconnection paradigms. It profoundly analyzes Indonesia as a welfare state from the disciplines of philosophy, law, social studies, etc. Fundamentally, the purpose of the Republic of Indonesia according to the founding fathers is to create social welfare. The aim of this state is legally and formally stipulated in some constitutional regulations. Unfortunately, Indonesia has not yet succeeded in creating the envisioned welfare. The failure of the welfare nation applied by the political and economic policy experts is due to the forceful hit of the economic crisis. It should remain a lesson to be learned as the failure to reach the Indonesian welfare state is due to the misuse of power, such as corruption, human rights violations, or violations of the law.

Indonesia's failure in creating the welfare state in the transcendental philosophy as envisioned by the founding fathers may be seen in the domain of philosophy. The Indonesian law is aimed basically to provide prosperity, protection, assurance, and justice to all people. However, the law in this country experiences what we call ideological poverty that substantially omits the intrinsic soul of the law itself. The cause is that the institution with the authority to dictate law has ignored the welfare state philosophical reference from the founding fathers.

The founding fathers’ philosophy on the welfare state is neither liberalism nor communism. Prophetic-transcendentally, the characteristic of the Indonesian legal welfare state concept can be defined in the conception that the “Belief in the Almighty God” principle is fundamental in order to bring welfare to Indonesians. Indonesia may achieve welfare if it understands the welfare state philosophy as perceived by the founding fathers.

## Declarations

### Author contribution statement

Khudzaifah Dimyati: Conceived and designed the experiments; Performed the experiments; Analyzed and interpreted the data; Wrote the paper.

Haedar Nashir: Conceived and designed the experiments; Analyzed and interpreted the data; Contributed reagents, materials, analysis tools or data; Wrote the paper.

Elviandri: Performed the Experiment; Analyzed and interpreted the data; Wrote the paper.

Absori: Performed the Experiments; Analyzed and interpreted the data; Contributed reagents, materials, analysis tools or data; Wrote the paper.

Kelik Wardiono: Performed the experiments; Contributed reagents, materials, analysis tools or data; Wrote the paper.

Arief Budiono: Performed the experiments; Analyzed and interpreted the data; Contributed reagents, materials, analysis tools or data; Wrote the paper.

### Funding statement

This research did not receive any specific grant from funding agencies in the public, commercial, or not-for-profit sectors.

### Data availability statement

Data included in article.

### Declaration of interests statement

The authors declare no conflict of interest.

### Additional information

No additional information is available for this paper.
